# Revealing the effect of a hidden dimension on slug spatial distributions in arable fields

**DOI:** 10.1098/rsos.250318

**Published:** 2025-09-17

**Authors:** Natalia B. Petrovskaya, Yadigar Sekerci Firat, John R. Ellis, Sergei Petrovskii

**Affiliations:** ^1^ School of Mathematics, University of Birmingham, Birmingham, UK; ^2^ Department of Mathematics, University of Hull, Hull, UK; ^3^ Department of Mathematics, Amasya University, Amasya, Amasya Province, Turkey; ^4^ Infectious Disease Epidemiology, Imperial College London, London, UK; ^5^ School of Computing and Mathematical Sciences, University of Leicester, Leicester, UK; ^6^ Peoples' Friendship University of Russia, Moscow 117198, Russian Federation

**Keywords:** grey field slug (*Deroceras reticulatum*), spatial distribution, patch formation, pesticide application

## Abstract

The movement and spatial distribution of the grey field slug (*Deroceras reticulatum*) in arable fields are investigated in ongoing interdisciplinary research motivated by the concept of targeted pesticide application in the population of slugs. The previous work suggested an explanation for the self-organized formation of a heterogeneous (patchy) spatial population distribution of slugs due to their density-dependent movement on the soil surface. This article reports new results of the study in which a vertical movement feature has been added to the baseline two-dimensional mathematical and computational model to see how it may contribute to the appearance and disappearance of slug patches in arable fields. A model of spatio-temporal dynamics of vertical movement is presented in the paper and correlation between spatial distributions of the slug population on and under the soil surface is investigated. It is explained how the probabilities of upward and downward movement in the new three-dimensional model impact the formation and stability of patchy spatial patterns overground and underground. It is suggested that the vertical movement of the slugs should be taken into account when evaluating the efficiency of pesticide application.

## 1. Introduction

The grey field slug, *Deroceras reticulatum* (Müller, 1774), is a common pest in many agricultural settings in Europe and North America, where it can cause considerable economic damage [1,2]. The application of pesticides is essential to control the population of slugs, but recent agricultural policies require a reduction in the amount of pesticides used in the population of slugs in arable fields [3]. Furthermore, the efficiency of pesticide application on slugs is not high: it was argued in [4] that, at best, molluscicides kill approximately 50% of the slug population and practitioners admit that the reasons behind the low efficacy of pesticides remain unclear to them [5].

To make the application of pesticide on slugs more effective, in [6], it has been proposed to take into account the strongly heterogeneous spatial distribution of slugs when the pesticide is applied. For several decades, terrestrial molluscs, including slugs, have been known to have heterogeneous spatial distributions [7]. The population of slugs in arable fields has spatial patches with a higher number of slugs interspersed with spatial subdomains with a much lower number of slugs, regardless of the size of the population [3,7–11]. Targeting control measures only in spatial domains with high slug densities (slug patches) can potentially reduce the amount of pesticide used in the slug population without making slug control less efficient.

The targeted application of pesticide can only be exploited when the mechanisms of slug patch formation are clear and the temporal dynamics of patches can be accurately predicted. Practitioners should be well informed about how fast slug patches are formed and how long they will exist, and these questions have been investigated in an interdisciplinary project involving biologists and applied mathematicians [12–15]. A novel individual-based model (IBM) was developed in the simulation of slug movement, where the model was parametrized from the experimental study [12,15]. The model agreed very well with the real-life data. Patch formation was observed in the model, and heterogeneous spatial distributions of slugs had the same characteristics (e.g*.* the degree of patchiness and the average size of patch) as those obtained in field observations.

Meanwhile, a two-dimensional model of slug movement developed in [12,15] (see also [16] for all technical details) cannot be considered complete because it does not take into account the vertical movement of slugs. An important feature of the slug population is that slugs move and feed on the ground only under suitable environmental conditions, e.g*.* if the weather is not too hot or dry. Otherwise, slugs go to soil cracks and sheltered cavities under the soil surface as their physiological response to harsher environmental conditions when soil temperatures rise and moisture falls [17].

The vertical movement of the slugs may have a crucial impact on the stability of the density patches. An important question to be answered in the design of a monitoring and control protocol for the targeted application of pesticides is the following. If a slug patch overground is destroyed by pesticides, can the patch re-emerge later from the slug population underground? Further investigation of this question is required before targeting pesticides in selected spatial domains can be offered, and in the present study, the IBM developed in the previous work has been extended to investigate how upward and downward movement of slugs may impact their heterogeneous spatial distribution overground and underground.

This article is organized as follows. In the next section, we briefly recall a two-dimensional model of slug movement developed in [12,15] to study the spatio-temporal dynamics of the overground slug population and explain how vertical movement can be incorporated into the model. In §3, we then analyse the vertical movement of the slugs to understand how the entire population is distributed between the overground and underground locations. An accurate prediction of the number of slugs overground and underground made in §3 allows one to conclude how the spatial distribution of slugs on the soil surface is related to their spatial distribution underground and will be discussed in §4. We then demonstrate in §5 how taking into account the vertical movement of the slugs allows one to explain the low efficacy of the application of pesticides. Conclusions and directions for future work are offered in §6 where we argue that vertical movement is crucial for understanding the spatio-temporal dynamics of the slug population.

## 2. Simulation of three-dimensional slug movement

**Figure 1.**
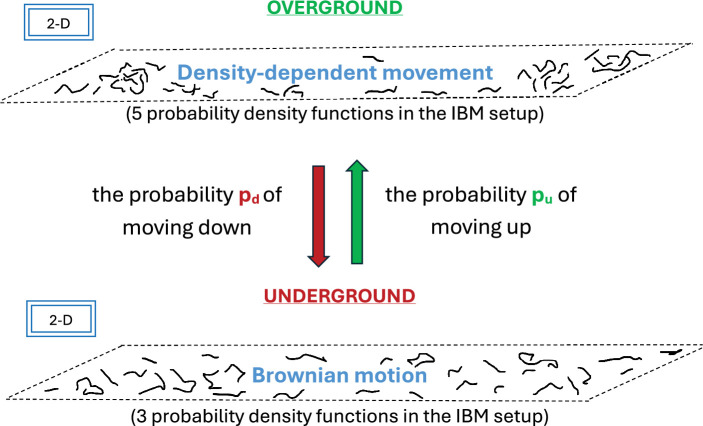
A sketch of the three-dimensional setup used in IBM simulation. It is assumed that all slugs underground are at the same depth, and the three-dimensional setup can be split into 2 two-dimensional setups where IBM rules are different (see further explanation in the text). Vertical movement of slugs is defined by the probabilities $p_{u}$ and $p_{d}$. Hypothetical trajectories of slugs are shown as black solid lines in the (x,y)-plane overground and underground.

Consider a population of N slugs overground (i.e. at depth z=0), where location $\left(x_{n}(t),y_{n}(t)\right)$ of the nth slug, n=1,2,…,N, changes with time, as defined by specific rules of their movement in the IBM setup [12,15] . Time progresses discretely with a constant increment Δt=1, i.e. we consider times t=1,2…,T in our model. Let us also have a population of P slugs underground, where we assume that all slugs in the underground population are at the same depth $z=z^{*}<0$ (figure 1). Hence, the population underground can be considered in the two-dimensional framework where the location of the pth slug, p=1,2,…,P, underground is projected onto the (x,y)-plane positioned at depth $z^{*}$. The locations $\left(x_{p}(t),y_{p}(t)\right)$, p=1,2,…,P also depend on time, but the movement of the slugs underground is defined by different rules in IBM. Overall, we split the entire three-dimensional setup into two coupled two-dimensional setups in our model for the sake of simplicity, where populations at the depth z=0 (overground) and $z=z^{*}$ (underground) interact with each other, as will be explained further in the text.

Since we are interested in spatial distributions of the population density overground and underground, the first step in our investigation is to define the spatio-temporal dynamics of the slug population, i.e. the position $\left(x_{n}(t),y_{n}(t)\right)$, where n=1,2,…,N (the population overground) and $\left(x_{p}(t),y_{p}(t)\right)$, p=1,2,…,P (the population underground) for any time t=1,2,…,T. In the previous work [15], where the population was considered overground only, standard movement characteristics quantified by relevant probability distributions were introduced in line with the classical IBM approach (e.g. [18]). For every slug in the population, the movement/rest time ratio, the turning angle and the step length were considered, each of them being quantified by a probability distribution. An essential feature of the model was the simulation of density-dependent movement of the slug population based on the results of the experimental study [3,13,14], and now we employ the same approach as in [15] to simulate slug movement overground.

The density-dependent movement of the population N at the depth z=0 is incorporated into the standard IBM by introducing two additional movement characteristics: a perception radius R≥0 (e*.*g. [19,20]) and the density threshold d. The movement of each slug to its next position at time t is then quantified as sparse if the density of the slugs $D_{l}$ within an individual’s perception radius is below the density threshold $D_{l}<d$. If the density of the slug $D_{l}$ is above the density threshold, $D_{l}>d$, then the movement of the slugs is considered dense at time t and other parameters and probability distributions are used to calculate the position of the slug at time t+1. Let $p_{m}$ be the average movement frequency, i.e. the ratio of the number of time steps in which slug movement is recorded to the total number of time steps registered in observations. For each slug overground at each time t, we assume that movement occurs when $p<p_{m}$, where the probability of movement p is drawn from a uniform distribution in the interval [0,1]. Importantly, $p_{m}$ is different for dense and sparse movement, that is,


(2.1) $$p_{m}=p_{m}^{s}\;\text{ if }\;D_{l}<d\;\text{ and }\;p_{m}=p_{m}^{d}\;\text{ if }\;D_{l}\geq d.$$

The density-dependent movement model has been parametrized using existing field data [12]. In our simulation, we consider the non-dimensional time,


(2.2) $$\begin{array}{lcr} & t=t^{*}/\Delta t^{*}, & \end{array}$$

where $t^{*}$ is the real time in minutes and $\Delta t^{*}=30$ min is a typical experimental time interval, so that Δt=1 in the model equates to 30 min. Based on the results of the field study, the value of the perception radius R was estimated to be between 0.2≤R≤2 m; correspondingly, in simulations, we use R=1 m. From available field data, the density threshold was roughly estimated as 0.25≤d≤100 slugs m${\;}^{-2}$, i.e. the movement is known to be density dependent when the density of the slugs is $D_{l}>100$ (in the above units) and density independent when the density is $D_{l}<0.25$ [12]. Consequently, in simulations, we use the density threshold value $D_{l}=50$ slugs m${\;}^{-2}$. The average movement frequencies $p_{m}$ that the slug movement data showed in the sparse and dense releases were evaluated in [12] as $p_{m}^{s}\approx 0.5$ for sparse movement and $p_{m}^{d}\approx 0.25$ for dense movement.

We then define the direction of the movement θ quantified by the turning angle, i*.*e*.* the angle made between the directions of the previous and the next steps. Based on the analysis of field data [12], the probability distribution for the turning angle is as follows. For slugs in the sparse movement, we use a Von Mises distribution:


(2.3) $$\begin{array}{lcr} & \rho (\theta )=\frac{\exp(\kappa \;\cos(\theta -\mu ))}{2\pi I_{0}(\kappa )}. & \end{array}$$

The analysis of the slug movement data provided the parameter values in (2.3) as μ=0 and κ=0.8 (see [12] for details). For slugs in dense movement, an approximation of the data by a Von Mises distribution yields the value κ≈0. Correspondingly, for the turning angle distribution, we use the uniform distribution,


(2.4) $$\begin{array}{lcr} & \rho (\theta )=\frac{1}{2\pi }, & \end{array}$$

which is the limiting case of the Von Mises distribution when κ=0.

We also need to define a probability distribution for the step length Δr. It was shown in [12] (see also Section 5.2.1 in [16] for all technical details) that the latter is best described by a half-normal distribution for both sparse and dense movement but with a different variance. We have


(2.5) $$\begin{array}{lcr} & \rho_{G}(\Delta r|0,\sigma^{2})=\frac{2}{\sqrt{2\pi \sigma^{2}}}\exp\left(-\frac{(\Delta r{)}^{2}}{2\sigma^{2}}\right), & \end{array}$$

where


(2.6) $$\sigma^{2}=\sigma_{s}^{2}\;\text{ if }\;D_{l}<d\;\text{ and }\;\sigma^{2}=\sigma_{d}^{2}\;\text{ if }\;D_{l}\geq d.$$

The distribution (2.5) has been fitted to the data to obtain $\sigma_{s}=0.105$ m for sparse movement and $\sigma_{d}=0.113$ m for dense movement over a given time interval of 30 min.

Given the position $\left(x_{n}(t),y_{n}(t)\right)$ of the nth slug, n=1,2,…,N, above ground at time t=0,1,2,…T−1, its position at time t+1 is simulated as


(2.7) $$\begin{array}{lcr} & \left(x_{n}(t+1),y_{n}(t+1)\right)=(x_{n}(t)+\Delta x,y_{n}(t)+\Delta y), & \end{array}$$

where


(2.8) $$\begin{array}{lrr} & \Delta x=(\Delta r)\;\cos(\theta ),\;\Delta y=(\Delta r)\;\sin(\theta ), & \end{array}$$

provided that the slug moves rather than remains at rest. The turning angle θ and the step length Δr are quantified by the corresponding probabilities, as explained above.

Consider now the population of slugs P at depth $z=z^{*}$. To the best of our knowledge, there is no empirical information at all on how slugs move inside the soil. We therefore have to make certain assumptions. Namely, we assume that in their movement below the surface slugs employ the simplest pattern of random movement, for which we consider the isotropic Brownian motion. In this case, the turning angle is distributed uniformly over the circle, cf. (2.4), as it is known to be a null model for the isotropic Brownian motion (e.g. [21]). For the step length Δr, we use (2.5) where the variance should be taken substantially smaller than the values $\sigma_{s}$ and $\sigma_{d}$ above to account for an intuitive expectation that slugs below the surface are likely to move slower than on the surface. In our simulations, we use a hypothetical value σ=0.05. We then calculate the position of each slug underground at time t+1 according to (2.7) and (2.8).

Finally, we have to introduce the rules of interaction between the populations overground and underground. We assume that the slug population is under stable environmental conditions, i.e*.* we investigate the case where both probabilities $p_{u}$ and $p_{d}$ of moving up and down, respectively, are constant. Let $x_{d}$ be a random number taken from a uniform distribution U(0,1) at time t. A slug belonging to the overground population will go underground at time t if $x_{d}<p_{d}$. Similarly, let $x_{u}$ be a random number taken from a uniform distribution U(0,1) at time t, then a slug belonging to the underground population will go overground at time t if $x_{u}<p_{u}$. We require that the planar position (x,y) of each slug does not change when the slug goes up or down. Furthermore, we assume that vertical movement of each slug is completed over one time step, i*.*e. slugs can only be found at depth z=0 or depth $z=z^{*}$. It is also important to note that the rules of vertical movement result in the change in the population overground and underground over time, as slugs move up and down at each time step.

Once the probability distributions have been defined in the model, the overall IBM consists of the following steps:

(1) For a given time t, t=0,1,2,…,T−1, consider the population overground and define whether each slug remains in the population or moves underground. Mark those slugs that will move underground (but without moving them).
(2) For the given time t, consider the population underground and define whether each slug remains in the population or moves overground. Mark those slugs that will move overground (but without moving them).
(3) Redefine the number of slugs in each population according to steps 1 and 2, that is, move the marked slugs from the overground to the underground, and vice versa accordingly.
(4) Compute the position $\left(x_{n}(t+1),y_{n}(t+1)\right)$ in the (x,y) plane for each slug in the new overground population N as defined by (2.1)–(2.8).
(5) Compute $\left(x_{p}(t+1),y_{p}(t+1)\right)$ for each slug in the new underground population P as defined by (2.4)–(2.8).
(6) Increase time by Δt=1 and repeat steps 1−5.

Computation in IBM starts from an initial condition, i*.*e. we have to define the position $\left(x_{k}(0),y_{k}(0)\right)$ at time t=0 for any slug overground or underground. We use a statistically uniform spatial distribution where the coordinates $x_{k}(0)$ and $y_{k}(0)$ of the kth slug at time t=0 are random numbers, each of them being drawn independently from a uniform distribution for the slug population overground and underground.

Given the parameters of the movement of the slugs, we implement the IBM procedure as described above. Individual-based simulations produce the position of all slugs in the population over and underground at any given time t. Those positions are then converted into the population density of slugs as follows. The spatial domain either overground or underground is divided into M=100 bins by dividing its linear size in both x and y into m=10 equal intervals. We make sure that the number of bins is the same for the overground and underground domains, i*.*e. the spatial domain overground is accurately mapped onto the spatial domain underground. We then calculate the number of slugs in each bin, and the population density ${\bar{u}}^{\left(i\right)}$ in the ith bin is introduced as


(2.9) $$\begin{array}{lcr} & {\bar{u}}^{\left(i\right)}=\frac{B_{i}}{A}, & \end{array}$$

where $B_{i}$ is the number of slugs in the ith bin and A is the area of the bin. We consider the population density to be constant in each bin and use the binned numbers ${\bar{u}}^{\left(i\right)}$, i=1,2,…,M to generate a continuous spatial distribution of the population density.

**Figure 2.**
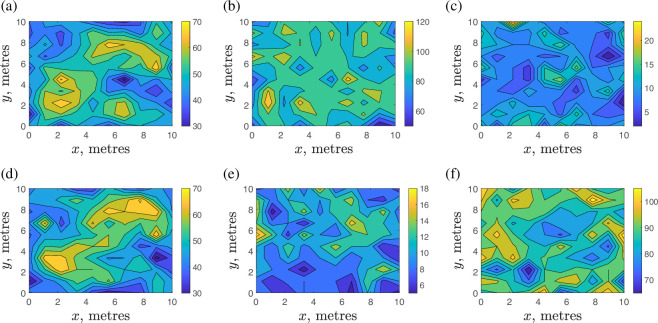
Spatial distribution of slugs overground and underground at the time T=10000 obtained for various probabilities $p_{u}$ (moving up) and $p_{d}$ (moving down): (a,d) $p_{u}=0.5$, $p_{d}=0.5$ (b,e) $p_{u}=0.9$, $p_{d}=0.1$, (c,f) $p_{u}=0.1$, $p_{d}=0.9$. For comparison, the upper row (a–c) shows the distributions overground and the lower row (d–f) shows the distributions underground. The total number of slugs in both populations overground and underground is $\Pi =10^{4}$. Slugs are distributed statistically uniformly with the population size Π/2 both overground and underground at the time t=0.

An example of our simulation is presented in figure 2 where we show spatial distributions of slugs overground and underground at the final time T=10000. Although the IMB simulation uses non-dimensional parameters, we keep the dimensional units for length so that the spatial scale of the emerging spatial patterns can be easily seen. We perform simulations in a spatial domain of 10×10 m which is consistent with field data on individual slug movement; see [12].

In the example shown in figure 2, the probabilities of each slug moving up or down are $p_{u}=const$ and $p_{d}=const$ over the entire time interval t∈[0,T]. Meanwhile, the choice of constant probabilities is various in each simulation in figure 2a–f to generate various spatial distributions at T=10000 as shown in the figure. We first consider $p_{u}=0.5$ and $p_{d}=0.5$ where the spatial distributions for that choice of the probabilities of vertical movement are shown in figure 2a (overground) and figure 2d (underground), respectively. The results of figure 2a confirm our previous conclusion in [12] that density-dependent movement overground results in formation of heterogeneous (patchy) spatial distributions. Visual inspection of figure 2d suggests that density-dependent movement of slugs on the surface also results in a similar heterogeneous spatial distribution of slugs beneath the surface.

It is important to note here that the time scale $t_{p}$ required for the formation of slug patches overground has been evaluated as $t_{p}∼10^{3}$ in [15] where the same parametrization (2.2) (i*.*e. $\Delta t^{*}=30$ min) has been used. When evolution of a statistically uniform spatial distribution into a heterogeneous spatial distribution occurs in the problem, the patches never become stationary due to the random nature of the slug movement. However, they roughly preserve their location and size after some time $t_{p}$, and the statistics of their spatial arrangement (as quantified by some statistical index) stop evolving at time $t_{p}∼10^{3}$.

Consider now a more extreme case of the probabilities of vertical movement $p_{u}=0.9$ and $p_{d}=0.1$ where the spatial distributions are shown in figure 2b (overground) and figure 2e (underground) at time $T=10^{4}$. There are still some overground slug patches, yet those patches are not projected underground where only random fluctuations in slug density appear (i*.*e. slug patches are not stable as they keep changing their location and size over time).

Another extreme choice of the probabilities of vertical movement is given by the case $p_{u}=0.1$ and $p_{d}=0.9$ presented in figure 2c (overground) and figure 2f (underground). It can be seen from the figure that there are no slug patches overground in this case, while the spatial distribution of slugs underground exhibits strong random fluctuations.

The results of figure 2 demonstrate that the spatial distributions of the slugs emerging on and below the soil surface in the 3D model are less predictable compared to the previous two-dimensional model in [12,15]. The presence/absence of slug patches on the surface where density-dependent slug movement is simulated in the three-dimensional models is not entirely defined by the IBM rules applied to the population overground, but depends also on the parameters $p_{u}$ and $p_{d}$. Thus, our next step is to quantify the extent to which the slug populations overground and underground are controlled by the probabilities $p_{u}$ and $p_{d}$ of vertical movement. That will be done in the next section.

## 3. Vertical movement of slugs with constant probabilities of vertical transition

In this section, we analyse the vertical movement of slugs to understand how the entire population is distributed between the overground and underground locations. Let $N_{t}$ denote the size of the slug population overground at time t. In order to make our model analytically tractable, we assume that the slug population is sufficiently large and therefore we can replace a random number of slugs going underground every time t by the expected number, i*.*e. we assume that the population fraction $p_{d}N_{t}$ will go underground at the next time t+1. Similarly, letting $P_{t}$ denote the population size of slugs underground at time t, we assume that the population fraction $p_{u}P_{t}$ will go overground in the next time step t+1. The vertical movement of the slugs is then described by the following model:


(3.1) $$\begin{array}{lcr} & \begin{array}{cc} N_{t+1}=(1-p_{d})N_{t}+p_{u}P_{t}, & \\ P_{t+1}=(1-p_{u})P_{t}+p_{d}N_{t}, & \end{array} & \end{array}$$

where $N_{t+1}$ and $P_{t+1}$ are overground and underground slug populations, respectively, at time t+1. We also require that the total slug population Π remains constant, i*.*e. we investigate the vertical movement of slugs outside the reproduction period:


(3.2) $$\begin{array}{lcr} & N_{t}+P_{t}=\Pi . & \end{array}$$

The difference equations (3.1) and (3.2) are solved at any time t=1,2…,T−1. At time t=0, we have the initial distribution of slugs overground and underground given by


(3.3) $$\begin{array}{lcr} & N_{0}=\alpha \Pi ,\;P_{0}=\beta \Pi , & \end{array}$$

where we require 0≤α≤1, 0≤β≤1 and α+β=1 to satisfy the condition (3.2).

The linear system (3.1)–(3.3) can be reduced to a single equation. Introducing a new variable


(3.4) $$\begin{array}{lcr} & W_{t}=p_{d}N_{t}-p_{u}P_{t}, & \end{array}$$

and performing a simple algebraic transformation of (3.1) gives


(3.5) $$\begin{array}{lcr} & W_{t+1}=CW_{t}, & \end{array}$$

where $C=1-p_{d}-p_{u}$. Equation (3.5) is augmented by the initial condition,


(3.6) $$\begin{array}{lcr} & W_{0}=(\alpha p_{d}-\beta p_{u})\Pi . & \end{array}$$

The solution to (3.5) and (3.6) is


(3.7) $$\begin{array}{lcr} & W_{t}=(\alpha p_{d}-\beta p_{u})(1-p_{d}-p_{u}{)}^{t}\Pi . & \end{array}$$

Let us define the ‘response’ function $F(W_{t})=CW_{t}$. Equation (3.5) has a single steady state $W_{t}^{*}=0$, which is stable if $|dF(W_{t}^{*})/dW_{t}|<1$. Since we have $dF(W_{t}^{*})/dW_{t}=C=1-p_{u}-p_{d}$ and the probabilities $p_{u}$ and $p_{d}$ are bounded by $0<p_{u}<1$ and $0<p_{d}<1$, the steady state is always stable. We note that the convergence to the steady state is different for various values of the parameter C as, apart from a degenerate case C=0 resulting in the steady-state solution immediately, we have monotone solutions for 0<C<1 and oscillatory solutions for −1<C<0.

Returning to the original system of equations (3.1)–(3.3), we obtain from the solution (3.7) that


(3.8) $$\begin{array}{lcr} & \begin{array}{cc} N_{t}=\frac{p_{u}+(\alpha p_{d}-\beta p_{u})(1-p_{d}-p_{u}{)}^{t}}{p_{u}+p_{d}}\Pi , & \\ P_{t}=\frac{p_{d}-(\alpha p_{d}-\beta p_{u})(1-p_{d}-p_{u}{)}^{t}}{p_{u}+p_{d}}\Pi , & \end{array} & \end{array}$$

where a stable steady state of the system (3.1)–(3.3) corresponds to $W^{*}=0$ and is given by


(3.9) $$\begin{array}{lcr} & \begin{array}{cc} N^{*}=\frac{p_{u}}{p_{u}+p_{d}}\Pi , & P^{*}=\frac{p_{d}}{p_{u}+p_{d}}\Pi . \end{array} & \end{array}$$

**Figure 3.**
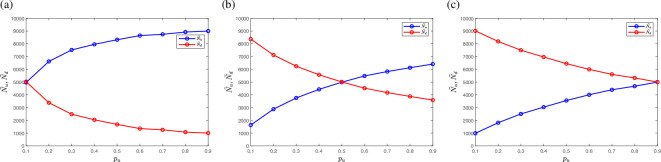
The average number of slugs overground (blue curve, blue open circles) and underground (red curve, red open circles) at the time *$T=10^{4}$* shown as a function of *$p_{u}$* for different values of the probability *$p_{d}$*: (a) *$p_{d}=0.1$*, (b) *$p_{d}=0.5$* and (c) *$p_{d}=0.9$*. The solid curves show the exact solution (3.9), while open circles show the average number of slugs obtained in IBM computation. The total number of slugs is *Π=10000*.

Some examples of steady state (3.9) are presented in figure 3 where we compare the exact solution with the overground and underground population size computed by direct IBM for various $p_{d}$ and $p_{u}$. In the latter case, the population size obtained at $T=10^{4}$ has been averaged over three runs. The purpose of comparing the exact solution (3.9) with the IBM results is to reveal the effect of stochasticity, because the solution (3.8) only shows the dynamics of the average values. It can be easily seen from the figure that the results of direct simulation are in very good agreement with the theoretical estimate (3.9) and the assumptions we have made in the development of our model are therefore well justified.

Consider the steady state $W^{*}=0$ of the equation (3.5). Given the result (3.7), the transition time $t^{*}$ required to approach the steady state from the initial distribution (3.6) can be evaluated from the convergence requirement,


(3.10) $$\begin{array}{lcr} & |W_{t}|<\epsilon , & \end{array}$$

where ϵ≪1, and (19) holds for any time $t>t^{*}$. Hence, we estimate $t^{*}$ from the following equation


(3.11) $$\begin{array}{lcr} & |(\alpha p_{d}-\beta p_{u})(1-p_{d}-p_{u}{)}^{t^{*}}|\Pi =\epsilon . & \end{array}$$

Since we have 0≤α≤1, 0≤β≤1, the initial condition (3.6) is bounded for $0<p_{u}<1$ and $p_{d}<1$ as


(3.12) $$\begin{array}{lcr} & |\alpha p_{d}-\beta p_{u}|<1, & \end{array}$$

and the maximum transition time required to converge with given accuracy ϵ from the initial state $W_{0}$ to the steady state $W^{*}=0$ is achieved for the maximum value of $|\alpha p_{d}-\beta p_{u}|$, i*.*e. when we have α=1, β=0 or α=0, β=1.

Consider the case α=1, β=0 when $N_{0}=\Pi$ and the entire slug population is initially overground (the opposite case α=0, β=1 will result in the same transition time). The estimate of the transition time $t^{*}$ computed from (3.11) when $p_{u}+p_{d}\neq 1$ and computed directly from (3.8) and (3.9) when $p_{u}+p_{d}=1$ is shown in table 1 for various values of $p_{u}$ and $p_{d}$ where we choose $\epsilon =10^{-4}$. It can be seen from the table that the transition time $t^{*}$ to the equilibrium between the overground and underground population is on the time scale of $t^{*}=1$ up to $t^{*}∼10^{2}$. Thus, we have $t^{*}\ll t_{p}$, i*.*e. the transition time $t^{*}$ to equilibrium is negligible compared to times $t_{p}$ over which slug patches are formed overground (see §2).

**Table 1.** The transition time to the steady state (3.9) for various probabilities of vertical movement $p_{u}$ and $p_{d}$ . The expression (3.11) is used to estimate the transition time where the parameters are taken as α=1, β=0, Π=10000 and $\epsilon =10^{-4}$ .

P_d_	0.1	0.2	0.3	0.4	0.5	0.6	0.7	0.8	0.9
**P**_**u**_									
**0.9**	1	7	11	15	19	26	35	51	82
**0.8**	7	1	7	11	15	19	26	35	51
**0.7**	11	7	1	7	11	15	19	26	35
**0.6**	14	11	7	1	7	11	14	19	25
**0.5**	19	14	11	7	1	7	11	14	19
**0.4**	25	19	14	10	7	1	7	10	14
**0.3**	33	24	18	14	10	7	1	7	10
**0.2**	47	32	24	18	13	10	7	1	7
**0.1**	72	45	31	23	17	13	10	6	1

Another important property of vertical movement is that the entire population is not ‘frozen’ in the equilibrium state (3.9). Any individual slug that can be found underground at time $t>t^{*}$ will then be found overground with probability $p_{u}$ at time t+1, while any individual slug overground will move underground with probability $p_{d}$ at the same time. Hence, the underground and overground populations keep exchanging a number of slugs at each time step even after approaching the equilibrium state. The average number of slugs $N_{f}^{u}$ going overground in the model (3.1) is $N_{f}^{u}=p_{u}P_{t}$, while the average number of slugs $N_{f}^{d}$ going underground is $N_{f}^{d}=p_{d}N_{t}$. We require the population fraction $N_{f}^{u}=N_{f}^{d}\equiv N_{f}$ at any time $t>t^{*}$ to maintain equilibrium between the two populations. Taking into account that $N_{t}$ and $P_{t}$ are defined by (3.9) in the equilibrium state, we obtain the following.


(3.13) $$\begin{array}{lcr} & N_{f}=\frac{p_{u}p_{d}}{p_{u}+p_{d}}\Pi . & \end{array}$$

In the next section, we will demonstrate how the presence of flux (3.13) in the system has a strong impact on the formation of slug patches in the overground and underground.

## 4. Results: comparison of spatial distributions of slugs overground and underground

Our aim in this section is to investigate how the spatial distributions overground and underground are similar to each other, depending on the probabilities of vertical movement $p_{u}$ and $p_{d}$. The standard statistical approach to analyse similarity between spatial distributions is to compute the (cross)correlation coefficient (e*.*g. see [22,23]). Consider the time t and let ${\bar{u}}_{ov}^{\left(i\right)}$ and ${\bar{u}}_{un}^{\left(i\right)}$ be the population densities in the ith bin (2.9) overground and underground, respectively. The correlation coefficient ρ between the spatial distributions overground and underground at the time t is given by


(4.1) $$\begin{array}{lcr} & \rho =\frac{\sum_{i=1}^{M}({\bar{u}}_{ov}^{\left(i\right)}-\mu_{ov})({\bar{u}}_{un}^{\left(i\right)}-\mu_{un})}{\sqrt{\sum_{i=1}^{M}({\bar{u}}_{ov}^{\left(i\right)}-\mu_{ov}{)}^{2}\sum_{i=1}^{M}({\bar{u}}_{un}^{\left(i\right)}-\mu_{un}{)}^{2}}}\;, & \end{array}$$

where $\mu_{ov}$ and $\mu_{un}$ are the sample means taken over all bins overground and underground, respectively. We have


(4.2) $$\begin{array}{lrr} & \mu_{ov}=\frac{1}{M}\sum_{i=1}^{M}{\bar{u}}_{ov}^{\left(i\right)},\;\;\;\mu_{un}=\frac{1}{M}\sum_{i=1}^{M}{\bar{u}}_{un}^{\left(i\right)}, & \end{array}$$

where M is the total number of bins.

The correlation coefficient is −1≤ρ≤1 where ρ>0 corresponds to correlated spatial distributions. Hence, we will have ρ>0 if density patches overground are projected underground as a result of vertical movement of slugs, where ρ≈1 will indicate almost identical patches overground and underground. On the other hand, two statistically uniform spatial distributions will have the correlation coefficient ρ≈0.

**Figure 4.**
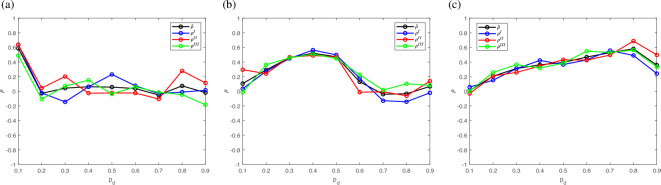
The correlation coefficient *ρ* is plotted against vertical movement down probability *$p_{d}$* for various vertical movement up probability *$p_{u}$*: (a) *$p_{u}=0.1$*, (b) *$p_{u}=0.5$*, (c) *$p_{u}=0.9$* for *$N=10^{4}$* slugs at *T=10000*. Each of the correlation coefficients *$\rho^{I}$* (blue line, open circles), *$\rho^{II}$* (red line, open circles) and *$\rho^{III}$* (green line, open circles) is obtained as a result of a single run of the IBM code, while the correlation coefficient *$\bar{\rho }$* (black line, open circles) is obtained by averaging *$\rho^{I}$*, *$\rho^{II}$* and *$\rho^{III}$* in (4.3).


An example of the correlation coefficient (4.1) between the spatial distributions overground and underground is shown in figure 4. The graphs in the figure are presented in time $T=10^{4}$ for various probabilities $p_{d}$ and three different values of probability $p_{u}$. Since slugs move randomly, the correlation coefficient is slightly different in each simulation of our IBM, and we also show the average correlation coefficient $\bar{\rho }$ computed as

(4.3) $$\begin{array}{lcr} & \bar{\rho }=\frac{1}{N_{R}}\sum_{k=1}^{N_{R}}\rho_{k}, & \end{array}$$

where $\rho_{k}$ is the correlation coefficient obtained in the kth simulation and $N_{R}$ is the number of stochastic realizations. In the example of figure 4, we have $N_{R}=3$, i*.*e. we run the IBM code three times. The correlation coefficients obtained as a result of each run are shown in the figure as graphs $\rho^{I}$, $\rho^{II}$ and $\rho^{III}$ along with the average correlation coefficient (4.3).

It follows from the graphs in figure 4 that the correlation coefficient ρ depends strongly on the choice of probabilities $p_{u}$ and $p_{d}$. Interestingly and rather counterintuitively, in most cases the functions $\rho (p_{u})$ and $\rho (p_{d})$ are distinctly non-monotonous (figure 4b,c), where $\rho (p_{d})$ is a nearly cyclic function in figure 4b. We note that the maximum correlation is achieved when those probabilities are approximately the same (cf. figure 2).

More examples of the nonlinear behaviour of the correlation coefficient ρ can be seen in table 2 where we show correlation coefficients for various probabilities $p_{u}$ and $p_{d}$. The results in figure 4 suggest that $N_{R}=5$ can be used as a number of runs sufficient to smooth out random oscillations in the correlation coefficient. Thus, each value $\rho (p_{u},p_{d})$ in table 2 has been obtained as a result of averaging (4.3) over $N_{R}=5$ runs (the bar in the notation is omitted for convenience). The dependence $\rho (p_{u},p_{d})$ arguably reflects the inherent complexity of the system, and below we analyse the results in table 2 based on the properties of our IBM model.

**Table 2.** The average correlation coefficient ρ between the spatial distribution of slugs overground and underground obtained for the various probabilities of vertical movement $p_{u}$ and $p_{d}$ at the time $T=10^{4}$. The total population is Π=10000 slugs (see §2 for the other parameters in IBM simulation).

P_u_	0.1	0.2	0.3	0.4	0.5	0.6	0.7	0.8	0.9
**P**_**d**_									
**0.9**	−0.02	−0.004	0.01	0.10	0.07	0.02	−0.03	0.08	0.36
**0.8**	0.07	0.003	−0.01	−0.002	−0.03	0.16	0.23	0.48	0.58
**0.7**	−0.05	0.001	−0.01	−0.1	−0.04	0.28	0.50	0.42	0.53
**0.6**	0.04	−0.01	−0.04	−0.03	0.13	0.58	0.57	0.52	0.47
**0.5**	0.06	0.05	0.02	0.15	0.47	0.50	0.44	0.42	0.39
**0.4**	0.06	0.01	0.1	0.48	0.52	0.52	0.50	0.41	0.36
**0.3**	0.04	−0.05	0.52	0.53	0.46	0.36	0.26	0.31	0.31
**0.2**	−0.03	0.53	0.50	0.32	0.29	0.32	0.21	0.24	0.21
**0.1**	0.59	0.35	0.26	0.20	0.10	0.17	0.11	0.13	0.01

First, we observe that the time $T=10^{4}$ when the correlation coefficient is computed is $T\gg t^{*}$, where $t^{*}$ is the transition time to steady state (3.9). Hence, the entire population has already been in dynamic equilibrium for a long time before the correlation coefficient is computed between the spatial distributions overground and underground at the time T. In the following, we investigate the two features of the steady state that have an impact on the correlation coefficient. The first is the size of the slug population as defined by the expressions (3.9). Let us introduce the ratio r of the population size overground $N^{*}$ to the population size underground $P^{*}$ in the steady state (3.9). We have


(4.4) $$\begin{array}{lcr} & r=\frac{N^{*}}{P^{*}}=\frac{p_{u}}{p_{d}}. & \end{array}$$

The ratio r of the populations is shown in table 3. Clearly, the population size underground is the same as the population size overground for the entries at the anti-diagonal in the table. It is also readily seen from (4.4) that the population size is $N^{*}<P^{*}$ for $p_{u}<p_{d}$ and $N^{*}>P^{*}$ for $p_{u}>p_{d}$. The comparison of results in tables 2 and 3 reveals that the maximum correlation between spatial distributions overground and underground occurs when the populations $N^{*}$ and $P^{*}$ do not differ in size significantly, that is, when their ratio is 0.8<r<2 (table 3). Since the ratio r of population sizes is entirely defined by the probabilities $p_{u}$ and $p_{d}$, we want to analyse the results based on the choice of these parameters. The following example illustrates the case where the probabilities of vertical movement are $p_{u}<p_{d}$.

**Table 3.** The ratio r (4.4) of the slug population overground and underground in the steady state (3.9). The parameters of IBM simulation are the same as in table 2.

P_u_	0.1	0.2	0.3	0.4	0.5	0.6	0.7	0.8	0.9
**P**_**d**_									
**0.9**	0.111	0.222	0.333	0.444	0.555	0.667	0.777	0.888	1
**0.8**	0.125	0.25	0.375	0.5	0.625	0.75	0.875	1	1.125
**0.7**	0.143	0.286	0.429	0.571	0.714	0.857	1	1.143	1.286
**0.6**	0.166	0.333	0.5	0.667	0.833	1	1.167	1.333	1.5
**0.5**	0.2	0.4	0.6	0.8	1	1.2	1.4	1.6	1.8
**0.4**	0.25	0.5	0.75	1	1.25	1.5	1.75	2	2.25
**0.3**	0.333	0.667	1	1.333	1.667	2	2.333	2.667	3
**0.2**	0.5	1	1.5	2	2.5	3	3.5	4	4.5
**0.1**	1	2	3	4	5	6	7	8	9

### 4.1. Example 4.1

Let the probabilities of moving up and down be $p_{u}=0.1<p_{d}=0.9$ and let the entire population be Π=10000 slugs. The population ratio r=1/9≈0.111 in table 3 indicates that the overground and underground populations have a very different size. The population overground is calculated from (3.9) as $N^{*}=0.1\Pi =1000$ slugs, while $P^{*}=0.9\Pi =9000$ slugs. The number $N^{*}$ of slugs is not sufficient to form density patches overground (figure 2c) as we have the average density of slugs as 10 slugs m${\;}^{-2}$ in the population overground, while the density threshold used in our IBM simulation is $D_{l}=50$ slugs m${\;}^{-2}$ (see §2 for details). Hence, there is a small statistically uniform population overground and a large statistically uniform population underground. Those spatial population distributions do not correlate as confirmed by the low correlation coefficient ρ=−0.02 in table 2.

Consider now the opposite case where the probabilities are $p_{u}>p_{d}$ and let us apply the same analysis as in the previous example.

### 4.2. Example 4.2

Let us have $p_{u}=0.9>p_{d}=0.1$ and the same entire population of Π=10000 slugs. The population ratio r=9 indicates that the population overground dominates significantly. The direct computation in (3.9) gives the populations overground and underground as $N^{*}=0.9\Pi =9000$ slugs, $P^{*}=0.1\Pi =1000$ slugs, respectively. As in the previous example, the populations have a very different size, but the population $N^{*}$ is now large enough to form density patches overground (figure 2b). The average density of slugs overground is 90 slugs m${\;}^{-2}$ which is greater than the density threshold $D_{l}=50$ slugs m${\;}^{-2}$. Meanwhile, the correlation coefficient is ρ=0.01 and the low correlation between the spatial distributions overground and underground suggests that the density patches overground may not be projected underground, which requires further investigation.

In order to explain the low correlation between the spatial distributions overground and underground in the above examples (especially in the example 4.2 above where patches are formed overground), we now look at another important feature of the steady state (3.9). We could see in §3 that there is a persistent population flux $N_{f}$ between the slug population overground and underground due to the stochastic nature of the slug movement, and the flux $N_{f}$ becomes constant in the equilibrium state as defined by (3.13). Let us introduce the scaled population fraction,


(4.5) $$\begin{array}{lcr} & f=\frac{N_{f}}{\Pi }. & \end{array}$$

The quantity f is shown for various probabilities of vertical movement $p_{d}$ and $p_{u}$ in table 4 and our analysis proceeds based on the results in the table.

**Table 4.** The population flux (4.5) in the steady state (3.9). The population fraction f exchanged between the slug population overground and underground at any time $t>t^{*}$ is shown for various probabilities of vertical movement $p_{u}$ and $p_{d}$. The parameters of IBM simulation are the same as in table 2.

P_u_	0.1	0.2	0.3	0.4	0.5	0.6	0.7	0.8	0.9
**P**_**d**_									
**0.9**	0.09	0.164	0.225	0.277	0.321	0.36	0.394	0.424	0.45
**0.8**	0.089	0.16	0.218	0.267	0.308	0.343	0.373	0.4	0.424
**0.7**	0.088	0.156	0.21	0.254	0.292	0.323	0.35	0.373	0.394
**0.6**	0.086	0.15	0.2	0.24	0.273	0.3	0.323	0.343	0.36
**0.5**	0.083	0.143	0.188	0.222	0.25	0.273	0.292	0.308	0.321
**0.4**	0.08	0.133	0.171	0.2	0.222	0.24	0.254	0.267	0.277
**0.3**	0.075	0.12	0.15	0.171	0.188	0.2	0.21	0.218	0.225
**0.2**	0.067	0.1	0.12	0.133	0.143	0.15	0.155	0.16	0.164
**0.1**	0.05	0.067	0.075	0.08	0.083	0.086	0.088	0.089	0.09

Consider first the probabilities of vertical movement $p_{u}<p_{d}$. It can be seen from the comparison of tables 3 and 4 that if $p_{u}<p_{d}$ then a large fraction of the population overground is replaced at each time $t>t^{*}$ by underground slugs, as illustrated by the following example.

### 4.3. Example 4.1 continued: the effect of population flux

Let the probabilities be $p_{u}=0.1$, $p_{d}=0.9$ and the entire population be Π=10000 slugs. The population fraction coming overground from the underground at each time step is $N_{f}=f\Pi =0.09\Pi$, while the total population overground is $N^{*}=0.1\Pi$. Hence, we have $N_{f}/N^{*}=0.9$, i*.*e. there are 90% of the population overground that come from the underground at each time $t>t^{*}$. Replacing a large fraction of the population overground by a statistically uniformly distributed population at each time t prevents patch formation. We also note that the same population fraction $N_{f}=0.09\Pi$ is brought from the surface to the underground at each time t, but it contributes as 10% of the underground population and does not have a lot of impact on the spatial distribution of the slugs there.

The above consideration confirms our previous conclusion that the populations overground and underground are not correlated when $p_{u}<p_{d}$ as the both populations can be thought of as statistically uniform spatial distributions (table 2).

Let us now investigate the case $p_{u}>p_{d}$, as in example 4.2. We have a larger population on the surface compared to the population beneath the soil, yet only a small fraction of it goes underground. The example below reveals a different mechanism for producing a low correlation between the two populations for probabilities $p_{u}>p_{d}$ compared to the previous case $p_{u}<p_{d}$.

### 4.4. Example 4.2 continued: the effect of population flux

Let us employ the parameters $p_{u}=0.9$, $p_{d}=0.1$, and let the total population be Π= 10 000 slugs. The population overground is $N^{*}=0.9\Pi$ and, as we have already mentioned in example 4.2, this population size is sufficiently large to form density patches (figure 2b). Patchiness of the spatial distribution underground will then depend on how many slugs are ‘projected’ by vertical movement from overground at each time. The population fraction from overground to underground at each time t remains the same as in the previous example, $N_{f}=f\Pi =0.09\Pi$. Hence, we have $N_{f}/N^{*}=0.1$, and only 10% of the population overground goes underground at each time $t>t^{*}$. This number of slugs is not sufficient to form a distinct projection of density patches of slugs overground to underground (figure 2e) which is indicated by the low correlation coefficient ρ=0.01 in table 2. It is worth noting here that the above conclusion is in good agreement with a previous analysis of patchiness made in [24,25] where the authors studied a patchy invasion model. In [25], it has been shown that patches in a spatial distribution of invasive species cannot be recognized if the spatial analysis is based on samples that include less than 15% of the population in the spatial domain.

The analysis of example 4.1 and example 4.2 reveals that the overground and underground populations are not correlated when $p_{u}>p_{d}$ because the number of slugs going underground is not sufficient to form a distinct projection of density patches overground; cf. tables 2 and 4. Overall, the spatial distributions overground and underground have the strongest correlation when the probabilities are $p_{u}\approx p_{d}$. In the latter case, the population size is $N^{*}\approx P^{*}$, the population overground is sufficiently large to form distinct patches and the number of slugs going underground is sufficiently large to project there the density patches formed overground.

To conclude this section, we note that the results presented in it have been obtained for probabilities that are widely varied, $0.1\leq p_{u,d}\leq 0.9$, and therefore, the range of biologically relevant values of $p_{u}$ and $p_{d}$ should be discussed before we proceed with our study. As we have shown in §3, regardless of the details of the initial distribution, the system is rapidly converging to a steady state with a certain constant number of slugs above and below the ground, this number depending on $p_{u}$ and $p_{d}$. Consider the case where one of the probabilities is small and the other is large, for instance $p_{u}=0.9$, $p_{d}=0.1$, or the other way around (figure 2). Once the system approaches the steady state, then at each time step, that is, every half an hour for our choice of the movement parameters, only 10% of the population travels up or down (table 4), which seems to be biologically reasonable. It is also important to note that 10% of the population moving vertically at each time step do not consist of the same slugs. At each time step, there will be mostly different slugs whose choice is dictated by the probability density function in our IBM as explained in §2. Furthermore, the number of slugs moving up or down is just 5% if both probabilities are small (e.g. $p_{u}=p_{d}=0.1$; table 4).

The only less realistic case is where both $p_{u}$ and $p_{d}$ are large. Consider, for example, $p_{u}=p_{d}=0.9$; in this case, almost half of the total population travels between layers at each time step (table 4). We assume that this intense exchange between populations above and below ground may occur under only exotic environmental and weather conditions, and therefore, this type of vertical movement will be rarely seen in arable fields. However, we include the above case in our conceptual model for the sake of completeness.

The study of dynamic equilibrium (3.9) allows one to understand whether or not density patches will be formed overground. In case that patches of the higher slug density have been formed, the analysis of the dynamic equilibrium can also help to come to a conclusion on whether those patches are projected underground. Meanwhile, the study of vertical movement of slugs in this section has been carried out under the assumption that the system does not experience any disturbance and the equilibrium (3.9) is preserved forever. In the next section, we extend our analysis to the case of a perturbed system when it moves to a new equilibrium state.

## 5. Results: application of pesticide

Efficient application of pesticides is a crucial requirement in most slug monitoring and control protocols; however, practitioners admit that the efficiency of application of pesticides on slugs is not high [4] and the reasons for the low efficiency of pesticides are not clear [5]. Hence, in this section, we investigate what can happen with the spatial distributions of slugs overground and underground when pesticide is applied to reduce the slug population. The application of pesticide can be considered as a perturbation that moves the population to a new equilibrium defined by the same expression (3.9) yet calculated for a new total population size $\Pi_{1}<\Pi$. Let the slug population overground and underground be in the equilibrium state $\left(N^{*},P^{*}\right)$ before the pesticide application procedure. Let also $0<\omega_{o}<1$ and $0<\omega_{u}<1$ be the remaining relative population overground and underground, respectively, after pesticide application. We assume that $\omega_{o}<\omega_{u}$ as the pesticide mostly kills slugs overground. After pesticide application, the overground and underground populations are $\tilde{N}=\omega_{o}N^{*}$ and $\tilde{P}=\omega_{u}P^{*}$, respectively, and a new total population size $\Pi_{1}$ is given by


(5.1) $$\Pi_{1}=\omega_{o}N^{*}+\omega_{u}P^{*}=\frac{\omega_{o}p_{u}+\omega_{u}p_{d}}{p_{u}+p_{d}}\;\Pi <\Pi .$$

The new distribution $\left(\tilde{N},\tilde{P}\right)$ is not a steady state of the population disturbed by pesticide application. Instead, it can be considered as a new initial condition (3.3), i*.*e. the system goes from state $\left(\tilde{N},\tilde{P}\right)$ to equilibrium state (3.9). The new initial distribution can be expressed as


(5.2) $$\begin{array}{lcr} & \begin{array}{cc} N_{0}\equiv \tilde{N}=\omega_{o}N^{*}=\frac{\omega_{o}p_{u}}{p_{u}+p_{d}}\;\Pi =\tilde{\alpha }\Pi , & \\ P_{0}\equiv \tilde{P}=\omega_{u}P^{*}=\frac{\omega_{u}p_{d}}{p_{u}+p_{d}}\;\Pi =\tilde{\beta }\Pi , & \end{array} & \end{array}$$

where $\tilde{\alpha }+\tilde{\beta }<1$ because the total population size has decreased. Under the assumption that the environmental conditions do not change, i*.*e. the probabilities of moving up and down remain the same as they were before pesticide application, the slug population reaches a new equilibrium $\left(N_{1}^{*},P_{1}^{*}\right)$ between the overground and underground population.

The new steady state $\left(N_{1}^{*},P_{1}^{*}\right)$ can be found by implementing the definition (3.9) subsequently to the original population size Π and the new population size $\Pi_{1}$ given by (5.1). We have


(5.3) $$\begin{array}{lcr} & \begin{array}{cc} N_{1}^{*}=\frac{p_{u}}{p_{u}+p_{d}}(\omega_{o}N^{*}+\omega_{u}P^{*})=\frac{\omega_{o}p_{u}^{2}+\omega_{u}p_{u}p_{d}}{(p_{u}+p_{d}{)}^{2}}\;\Pi , & \\ P_{1}^{*}=\frac{p_{d}}{p_{u}+p_{d}}(\omega_{o}N^{*}+\omega_{u}P^{*})=\frac{\omega_{o}p_{d}p_{u}+\omega_{u}p_{d}^{2}}{(p_{u}+p_{d}{)}^{2}}\;\Pi . & \end{array} & \end{array}$$

It follows immediately from (5.3) that the spatial distribution of the slugs after the application of pesticides depends on the probabilities of vertical movement $p_{u}$ and $p_{d}$. It is also important to note that the transition from the initial distribution (5.2) to the steady state (5.3) happens over time $t^{*}∼10^{1}$, as explained in §3. Rapid transition to the new steady state $\left(N_{1}^{*},P_{1}^{*}\right)$ accompanied by intense re-dislocation of slugs may result in wrong conclusions about a pesticide application procedure, as demonstrated below.

Consider first the case where the probability of moving upward is essentially smaller than the probability of moving downward. If the probabilities are $p_{u}\ll p_{d}$ then a large number of slugs are located underground. A fraction of those slugs will go overground after pesticide application to move the population to a new steady state defined by the new total population size $\Pi_{1}$. The redistribution of slugs underground and overground after pesticide application is illustrated by the following example.

### 5.1 Example 5.1

Let the probabilities of moving upward and downward be $p_{u}=0.1$ and $p_{d}=0.9$, respectively, and let the original population size be Π=10000 slugs. The original steady state is $N^{*}=0.1\Pi =1000$ slugs overground and $P^{*}=9000$ slugs underground. Since we have $p_{u}\ll p_{d}$, the original population overground does not have a distinctly patchy spatial distribution as shown in figure 2c; see also discussion in §4. Let $\omega_{o}=0.3$ and $\omega_{u}=0.9$, then the population overground becomes $\tilde{N}=0.3N^{*}=300$ slugs after pesticide application, while the population underground becomes $\tilde{P}=0.9P^{*}=8100$ slugs. The entire population is $\Pi_{1}=8400$ slugs after pesticide application and the spatial distributions overground and underground do not have high density patches.

However, the distribution $\left(\tilde{N},\tilde{P}\right)$ is not in a steady state and therefore a new steady state (5.3) will appear as $N_{1}^{*}=0.1\Pi_{1}=810$ slugs overground and $P_{1}^{*}=0.9\Pi_{1}=7290$ slugs underground after a very short time $t^{*}∼10^{1}$ (table 1). New slugs arriving from underground over a short transition period before the population approaches the new equilibrium will be distributed statistically uniformly over the spatial area as the original spatial distribution underground is statistically uniform.

As the new steady state is achieved very rapidly, practitioners returning to the field to evaluate the results of a pesticide application procedure may come to the false conclusion that only a small number of slugs were removed. We note that the transition $t^{*}∼10^{1}$ is on the time scale of several hours, given the time unit Δt=30 min that we use in the parametrization of the model (see §2). Meanwhile, conclusions about the efficiency of pesticide application are often made based on the results of a monitoring procedure that usually takes several days (or weeks, sometimes) [3]. Let us introduce the efficiency of pesticide application $e_{a}$ as follows:


(5.4) $$\begin{array}{lcr} & e_{a}=\frac{N^{*}-\tilde{N}}{N^{*}}100\%, & \end{array}$$

under the assumption that a monitoring protocol is designed to deal with the overground population only [3]. Substituting the values $N^{*}=1000$ and $\tilde{N}=300$, we obtain $e_{a}=70\%$ in example 5.1. Consider now an estimate of pesticide efficiency based on quantity $N_{1}^{*}$ as follows:


(5.5) $$\begin{array}{lcr} & e_{f}=\frac{N^{*}-N_{1}^{*}}{N^{*}}100\%, & \end{array}$$

where $N^{*}$ and $N_{1}^{*}$ are given by (3.9) and (5.3), respectively. Estimate (5.5) assesses the population size overground in the new equilibrium state in the situation when practitioners cannot differ between $\tilde{N}$ and $N_{1}^{*}$. Using (5.5) gives us a very wrong answer in example 5.1: since $N^{*}=1000$ and $N_{1}^{*}=810$, we obtain $e_{f}=19\%$, in contrast to the actual high efficiency of $e_{a}=70\%$.

Next, we consider the probabilities $p_{u}\approx p_{d}$. There is a strong correlation between spatial distributions overground and underground when the probabilities of going upward and downward are approximately the same (table 2). If condition $p_{u}\approx p_{d}$ persists for a relatively long time, higher density patches will be present in both overground and underground populations, as discussed in §4. After pesticide application, patchy spatial distributions will survive underground and emerge overground quickly when the system goes to a new steady state (5.3). The results of pesticide application in this case are illustrated by the following example.

### 5.2. Example 5.2

Consider the probabilities $p_{u}=0.5$, $p_{d}=0.5$ and the original population size Π=10000 slugs. The original steady state (3.9) is $N^{*}=0.5\Pi =5000$ slugs overground and the same number of $P^{*}=5000$ slugs underground. Density patches present in both overground and underground slug populations as shown in figure 2a,d.

Let us again assume, as in example 5.1, that 70% of the overground slugs and 10% of the underground slugs are removed from the population by application of pesticides. The population overground becomes $\tilde{N}=0.3N^{*}=0.15\Pi =1500$ slugs, while the population underground is now $\tilde{P}=0.9P^{*}=0.45\Pi =4500$ slugs. The entire population after pesticide application is $\Pi_{1}=0.15\Pi +0.45\Pi =0.6\Pi =6000$ slugs.

Starting from the distribution $\left(\tilde{N},\tilde{P}\right)$*,* the population makes rapid transition to a new equilibrium state (cf. transition times in table 1). The new steady state is given by the $N_{1}^{*}=0.5\Pi_{1}=3000$ slugs overground and the $P_{1}^{*}=0.5\Pi_{1}=3000$ slugs underground. As in the previous example, the effect of pesticide on the underground population is twofold: pesticide application removes 10% of the original population underground and also forces a large fraction $((\tilde{P}-P_{1}^{*})/P^{*})100\%=30\%$ of the original slug population underground to move overground rapidly. Again, the rapid emergence of overground slug density patches may lead practitioners to a false conclusion about the inefficiency of pesticide application. In the present example, the actual pesticide efficiency (5.4) is $e_{a}=70\%$, while a false estimate (5.5) gives $e_{f}=40\%$. The difference between them is not as striking as in Example 5.2; however, the reappearance of slug density patches that takes place in this case may reinforce the incorrect perception that the slug population is resistant to the pesticide.

Meanwhile, the above scenario can be favourable in a slug control routine, as it appeals to the targeted use of pesticide on higher density patches. After reappearance of the slug patches, the targeted application of pesticides in those spatial subdomains can be repeated. The double application of pesticide on the slug patches alone may require a smaller total amount of pesticide compared to its single application throughout the arable field and will also have a higher efficiency [6]. Although the topic of targeted use of pesticide requires further careful study, we believe it deserves further discussion in light of the results presented in §4 and 5.

Finally, consider the case where the probability of moving up is essentially higher than the probability of moving down, $p_{u}\gg p_{d}$. The spatial distribution of the overground slugs is patchy in this case, but the spatial distribution of the underground slugs does not have patches of higher density; see figure 2b and the explanation in §4. Slug patches will be destroyed overground by the application of pesticides, and redistribution of slugs will only result in a relatively small number of slugs moving overground. The effect of pesticide application in this case is illustrated by the following example.

### 5.3. Example 5.3

Let the probabilities of vertical movement be $p_{u}=0.9$, $p_{d}=0.1$ and the original population size be Π=10000 slugs. The original steady state (3.9) is $N^{*}=0.9\Pi =9000$ slugs overground and $P^{*}=0.1\Pi =1000$ slugs underground. Density patches have been formed overground, yet they are not projected underground, as shown in figure 2b,e.

Consider the baseline case $\omega_{o}=0.3$ and $\omega_{u}=0.9$ (cf. examples 5.1 and 5.2). After pesticide application, the population overground becomes $\tilde{N}=2700$ slugs and the population underground becomes $\tilde{P}=900$ slugs, so the entire new population becomes $\Pi_{1}=3600$ slugs. The new steady state (5.3) is given by the $N_{1}^{*}=0.9\Pi_{1}=3240$ slugs and the $P_{1}=0.1\Pi_{1}=360$ slugs. Hence, the estimate (5.5) gives $e_{f}=64\%$ which is close to the actual pesticide efficiency (5.4) $e_{a}=70\%$.

## 6. Conclusions

In the present paper, we have incorporated vertical movement into an individual-based model developed earlier to study the spatio-temporal dynamics of grey field slugs in arable fields [12,15]. Our study has been motivated by the concept of targeted pesticide application, already discussed in our previous work, where the two-dimensional IBM has been developed to explain the formation of spatial subdomains with the higher density of the slug population (slug patches) appearing as a result of density-dependent movement of slugs on the soil surface. In the present study, it has been revealed that taking into account vertical movement of slugs will result in a different spatial distribution of the slug population in comparison with the two-dimensional model. An important conclusion arising from our work is that while density-dependent movement on the soil surface is responsible for formation of a heterogeneous density distribution of slugs, if density-dependent movement overground is complemented by a high probability of moving down, then slug patches will not be formed.

In the paper, it has been argued that the probabilities $p_{u}$ and $p_{d}$ of the upward and downward movements, respectively, are the key characteristics of the three-dimensional slug movement. To the best of our knowledge, those parameters have usually been neglected by practitioners, and no information on them is available from field and laboratory studies. We hypothesize that, under typical weather conditions, it is likely that both $p_{u}$ and $p_{d}$ are small (e.g. $p_{u},p_{d}=0.1$ or so) since slugs do not have a strong motivation to move in the vertical direction and do it only occasionally. Under this hypothesis, if normal weather conditions persist for a relatively long time, then $p_{u}\approx p_{d}$ and slug patches will be present both in the population overground and underground, as discussed in §4. In that case, the targeted use of pesticide followed by repeated application of pesticide on reemerged patches will yield very good results, as it will remove a large fraction of the slug population from the field, as demonstrated in our model.

Furthermore, any significant change in the weather conditions will result in a rapid redistribution of the entire slug population. In this case, the assumption of both $p_{u}$ and $p_{d}$ being small does not apply anymore. If the ‘extreme’ weather conditions dictate that most of the slug population should go down the soil surface, i*.*e. the probabilities of vertical movement become $p_{d}\gg p_{u}$, then slug patches will rapidly disappear both overground and underground (cf. the results in [26] where the authors concluded based on their experimental study that slug patches that are stable under typical soil moisture conditions revert to a random distribution when the soil moisture is unusually high). Hence, the absence of patches overground may indicate that almost the entire slug population is located underground. In §5, it was argued that the above case may require multiple applications of pesticides across the arable field. On the other hand, the case where most slugs can be found overground, i*.*e. $p_{u}\gg p_{d}$, would require just a single targeted application of pesticide to completely destroy slug patches. If practitioners can identify the case $p_{u}\gg p_{d}$ from field observations, that situation will offer potentially large savings as the amount of pesticide required for its application in slug patches alone is much smaller compared to the uniform spread of the pesticide across the entire field [6].

In summary, our results demonstrate that vertical movement of the slug population is important and should be further investigated by applied biologists in both laboratory and field experiments to estimate the probabilities of upward and downward movement depending on weather conditions such as soil temperature and moisture. In case vertical movement of slugs is not taken into account, monitoring only the slug population overground can lead to a very imprecise estimation of the total slug population in the field. Meanwhile, if the probabilities $p_{u}$ and $p_{d}$ of vertical movement can be estimated for given weather conditions and the number of slugs overground is evaluated in the monitoring procedure, then the conclusion about the entire slug population can be made from equation (3.9). That, in turn, will allow practitioners to make an optimal decision about a pesticide application routine.

The model presented in this paper explains well the presence/absence of slug patches on the soil surface and also suggests a feasible explanation of the apparent low efficiency of pesticide application. Meanwhile, the setup we have considered is not entirely realistic, and further development of the model is required. First, the assumption of Brownian motion of the slugs underground should be carefully checked in field experiments. The parametrization of the underground movement of slugs (Brownian or otherwise) should be made based on experimental data, as was done for density-dependent movement over the ground in our previous work [12,15]. Slower or faster movement under the soil surface can result in a different conclusion about the correlation between spatial distributions overground and underground. Furthermore, we note that pesticide application during the reproductive season can lead to a different spatial distribution of the slugs as the conservation law (3.2) no longer holds, and the model (3.1)–(3.3) should therefore be revisited.

Another open question that our work leaves is dealing with time-dependent probabilities $p_{u}(t)$ and $p_{d}(t)$. This is a complex task as the probabilities of vertical movement can become time-dependent functions over a short period of time, e*.*g. if the diurnal cycle is considered, as well as a long period of time, e*.*g. if there is a long-lasting period of rapid changes in the weather conditions. For example, summer weather characterized by dry sunny days interspersed with heavy rain days throughout the summer will result in the situation where the system (3.1)–(3.3) does not approach the steady state (3.9) due to persistent perturbation. Thus, the transition from one nonstationary solution (3.7) to another in the model (3.1)–(3.3) and, more generally, the consideration of the probabilities of vertical movement as time-dependent functions is important and should be reserved as a topic for future work.

## Data Availability

Data and relevant code for this research work are stored in GitHub [27] and have been archived within the Zenodo repository [28].
